# An investigation into the critical ingredients of intensive support teams for adults with intellectual disabilities who display challenging behaviour

**DOI:** 10.1192/bjb.2023.94

**Published:** 2025-02

**Authors:** Lucretia Thomas, Brynmor Lloyd-Evans, Louise Marston, Angela Hassiotis

**Affiliations:** 1 Homerton University Hospital, London, UK; 2 University College London, London, UK

**Keywords:** Intellectual disability, intensive support teams, crisis care, challenging behaviour, community mental health teams

## Abstract

**Aims and method:**

NHS England recommends the commissioning of intensive support teams (ISTs) to provide effective support to people with intellectual disability (ID) when in crisis. However, there is a paucity of evidence regarding how these services should be organised. This exploratory secondary analysis of data from the IST-ID study aimed to investigate IST characteristics that relate to clinical outcomes. The primary outcome was mean change in the total score on the Aberrant Behavior Checklist and its subscales.

**Results:**

A measure of mental illness severity was the only variable associated with our primary outcome of reduction in challenging behaviour. Accommodation type, affective status and gender were associated with the subdomains of irritability, hyperactivity and lethargy in unadjusted and adjusted analyses.

**Clinical implications:**

Our findings indicate that variation in clinical outcomes is influenced by individual rather than organisational factors. Further research on the theoretical fidelity of the IST-ID model is needed.

Intellectual disability is defined as an impairment in intellect and adaptive functioning that begins during the neurodevelopmental period and persists throughout life.^1,2^

The National Institute for Health and Care Excellence (NICE) recommends that individuals with intellectual disabilities who are in crisis due to their mental health or behaviour receive support from a multidisciplinary intensive support team (IST).^3^ ISTs aim to reduce the occurrence and length of in-patient admissions by providing crisis care, in-reach support within in-patient units to facilitate discharge, and positive behaviour support in the community.^3–5^

NHS England's recommendations for ISTs include the provision of 24/7 face-to-face crisis support, multidisciplinary support delivered by specialists in the management of challenging behaviour, and integration between ISTs and specialist community teams that deliver routine care.^6,7^ There is little evidence regarding which service-level or individual participant characteristics of ISTs are associated with effective service delivery, whereas this is better established for crisis services for the general adult population and for older adults.^8–10^ This information is important in guiding the commissioning of services.^4,5^

The Intensive Support Teams for Adults with Intellectual Disabilities and Challenging Behaviour (IST-ID) national study investigated service-level characteristics and individual patient outcomes in existing ISTs.^4,5^ Stage 1 was a cross-sectional study that surveyed 73 ISTs in England and identified two types of such service provision: independent, where the IST is separate from the local community intellectual disability service, and enhanced provision, where the IST is integrated within it.^4^ Stage 2 was a cohort study comparing clinical outcomes between the two IST types at baseline and 9-month follow-up. The study did not find any differences in levels of challenging behaviour or any other secondary outcomes between the two types of IST provision, neither did it show significant cost differences. It concluded that local variations in need may well determine which model is adopted, in the absence of other requirements, including model fidelity.^5^

The present study complements the previous project by exploring how individual and service characteristics relate to clinical outcomes, generating hypotheses about potential critical components of IST care. This is very important because it brings into consideration the theoretical fidelity of ISTs, opening the discourse on the intervention theory behind such teams in the community care of people with intellectual disabilities.

## Aims

To investigate whether IST service-level characteristics relate to reduction of challenging behaviour measured by a validated instrument through the secondary analysis of data collected in the IST-ID study.

## Method

Data from participants who had enrolled in stage 2 of the IST-ID study were included in this secondary analysis. In stage 2, a random sample of ISTs was selected out of the 73 ISTs that had taken part in stage 1 of the study.^4,5^ To be included in stage 2, the IST must have been operating for at least 12 months, funded for the duration of the study and offering intensive support to adults with mild to profound intellectual disabilities.^5^

Full details of the procedures involved in data collection in the two stages of the IST-ID study can be found in the relevant publications.^4,5^

One individual (L.T.) reviewed all items from the survey administered in stage 1 of the IST-ID study to identify the most clinically important and cross-referenced them against published standards for ISTs.^3,11,12^ The results of this initial screening were reviewed by A.H., resulting in a shortlist of 27 items. This shortlist of items was discussed in a team meeting with all authors, when these were further refined and items that were considered to be less clinically relevant were removed (the longlist of items is provided in the supplementary material available at https://dx.doi.org/10.1192/bjb.2023.94). This resulted in a list of 16 items. We then combined some categories to produce binary or categorical variables with fewer categories. Finally, six items were removed owing to a lack of variation where all ISTs fell into one category, giving rise to a final list of ten service-level characteristics. This final list of variables is shown in Table 1.

**Table 1:** List of service-level variables identified from the IST-ID national survey

Service-level variable	Definition	Categories
Working hours	Whether the IST operates for 7–8 h per day, Monday to Friday, or has extended working hours	Normal or extended working hours
Psychiatry	Whether a psychiatrist is present in the multidisciplinary team	Yes or no
Criminal justice system	Whether the IST accepts patients who are either in contact with or who are at risk of coming into contact with the criminal justice system	Yes or no
Exemptions	Whether the IST operates any exemptions or exclusions in relation to accepting referrals	Yes or no
Source of referrals	Categorical variable representing the sources from which the IST accepts referrals	Accepts referrals from a community intellectual disability team but no other sources Does not accept referrals from a community intellectual disability team but does accept referrals from other sources Accepts referrals from a community intellectual disability team and from other sources
Target response time	Whether the IST has a target response time within which to commence an assessment in person	Yes or no
Functional assessment	Whether the IST offers functional assessments of challenging behaviour	Yes or no
Activities of daily living	Whether the IST offers the assessment of, training for or support with ADLs	Yes or no
Care coordination	Whether the IST offers care coordination	Yes or no
Frequency of visits	The average frequency of visits	More than once a week Once a week Other

IST-ID, Intensive Support Teams for Adults with Intellectual Disabilities and Challenging Behaviour; ADLs, activities of daily living.

The primary outcome was the mean change in challenging behaviour, measured by the total score on the Aberrant Behavior Checklist-Community version 2 (ABC-C) from baseline to 9-month follow-up.^13^ The secondary outcomes were the mean changes in each of the ABC-C subscale scores within the same time frame.

The scores from other validated questionnaires at baseline and demographic data were included in the multilevel linear regression models as covariates. The validated questionnaires included the affective/neurotic disorder and psychotic disorder subscales of the Psychiatric Assessment Schedule for Adults with Developmental Disabilities (PAS-ADD) Checklist, the Short Adaptive Behavior Scale (SABS) as a measure of adaptive functioning, the Threshold Assessment Grid (TAG) as a measure of clinical risk^14–16^ and the presence of attention-deficit hyperactivity disorder and/or autism spectrum disorder. Demographic data included age group (18–24 and ≥25 years), ethnicity (White, Black, Asian and minority ethnic), gender and accommodation type.

### Analysis of the primary and secondary outcomes

All statistical analyses were completed using Stata version 17 MP for Windows. We used descriptive statistics to describe the services and population.

Multilevel linear regression was used to model the effect of service-level characteristics on the mean change in ABC-C total score from baseline to 9-month follow-up, while controlling for covariates. These analyses were repeated for each of the secondary outcomes.

To ensure that the assumptions of multilevel linear regression were met, the residuals were plotted on a histogram and a standardised normal probability plot to assess for normality and were plotted against the predicted values to assess for homoscedasticity.

These analyses were completed as a series of three steps. In step 1, separate models were constructed for each of the service-level variables identified in the screening process described above. Within each model, the change in ABC-C total or ABC-C subscale score from baseline to 9-month follow-up was the dependent variable, the service-level variable was included as a fixed-effect independent variable. The purpose of this step was to identify which of the service-level variables had *P* < 0.10 and should therefore be included in the final model in step 3.

In step 2, the participant-level variables were included as fixed-effects independent variables.

In step 3, the model was constructed as in step 2 with the addition of the service-level variables identified in step 1 as fixed-effects independent variables. The purpose of step 3 was to determine whether the addition of the service-level variables affected the statistical significance of the model.

### Ethics and data protection

This secondary analysis of data from the IST-ID study involved the processing of anonymised data from stages 1 and 2 of study, of which A.H. is the guarantor. These data were stored and processed on a password-protected desktop computer at University College London, Division of Psychiatry, in compliance with General Data Protection Regulation (GDPR) policy and the Data Protection Act 2018. All data were kept strictly confidential.

The IST-ID study was performed in accordance with the Declaration of Helsinki. The Health Research Authority reviewed and approved the study and all amendments (substantial and non-substantial). Ethical approval for the IST-ID study was granted by the London Bromley Research Ethics Committee (reference 18/LO/0890). Further ethical approval was not required for the current study since it involved the secondary analysis of existing anonymised data obtained in the IST-ID study.

Consent of study participants to use their data was covered in the main ethical application for review. Outcomes of the analysis do not allow re-identifying participants and we did not transfer data to facilities outside of the UCL Division of Psychiatry.

## Results

Data regarding 226 participants across 21 ISTs were analysed. Tables 2 and 3 present service- and individual-level characteristics. In all the multilevel linear regression analyses, the residuals were normally distributed and there was no evidence of heteroscedasticity.

**Table 2:** Baseline characteristics of the included intensive support teams (ISTs)^a^

Characteristic	*n/N*	%
Working hours		
Normal working hours	5/21	23.81
Extended working hours	16/21	76.19
Psychiatry		
Yes	10/21	47.62
No	11/21	52.38
Criminal justice system		
Yes	20/21	95.24
No	1/21	4.76
Exemptions		
Yes	17/21	80.95
No	4/21	19.05
Source of referrals		
Accepts referrals from a community learning disability team but no other sources	4/21	19.05
Does not accept referrals from a community learning disability team but does accept referrals from other sources	2/21	9.52
Accepts referrals from a community learning disability team and other sources	15/21	71.43
Target response time		
Yes	12/21	57.14
No	9/21	42.86
Functional assessment		
Yes	19/21	90.48
No	2/21	9.52
Activities of daily living		
Yes	18/21	85.71
No	3/21	14.29
Care coordination		
Yes	17/21	80.95
No	4/21	19.05
Frequency of visits		
More than once a week	7/20	35.00
Once a week	7/20	35.00
Other	6/20	30.00

a. See Table 1 for clarification of the characteristics (service-level variables).

**Table 3:** Baseline characteristics of the included participants

	Mean or *n/N*	s.d. or %
**Demographic characteristics**		
Female gender	71/226	31.42%
Age ≥25 years	151/226	66.81%
Living status		
Family home	68/225	30.22%
Adult social care	141/225	62.67%
Living independently	16/225	7.11%
White ethnicity	181/226	80.09%
SABS score	52.44	24.03
**Clinical characteristics**		
Has one or more physical health problems	137/226	60.62%
Has ADHD and/or ASD	160/226	70.80%
TAG score	13.75	4.63
Meets PAS-ADD affective disorder threshold	48/226	21.24%
ABC-C score		
Total score	63.15	33.23
ABC-C irritability subscale score	20.24	10.70
ABC-C lethargy subscale score	14.31	9.23
ABC-C hyperactivity subscale score	18.76	11.96
ABC-C inappropriate speech subscale score	4.52	4.08

SABS, Short Adaptive Behavior Scale; ADHD, attention-deficit hyperactivity disorder; ASD, autism spectrum disorder; TAG, Threshold Assessment Grid; PAS-ADD, Psychiatric Assessment Schedule for Adults with Developmental Disabilities Checklist; ABC-C, Aberrant Behavior Checklist-Community version 2.

The univariate analysis conducted in step 1 demonstrated that working hours was the only variable found to be significantly associated with a change in ABC-C score (*P* < 0.1) (Supplementary Table 1).

Table 4 presents the results of the multivariable analyses in steps 2 and 3 for the primary outcome, change in ABC-C total score from baseline to 9-month follow-up. Results of the multivariable analyses in steps 2 and 3 for the secondary outcomes are shown in Supplementary Tables 2–6.

**Table 4:** Multivariable analyses in steps 2 and 3, for the primary outcome, where change in ABC-C total score from baseline to 9-month follow-up was the dependent variable

	Step 2		Step 3	
Characteristic	Coefficient	95% CI	Coefficient	95% CI
PAS-ADD affective positive	11.88	0.22 to 23.54	11.38	−0.21 to 22.97
PAS-ADD psychosis positive	−7.50	−28.65 to 13.65	−7.61	−28.68 to 13.45
SABS score	−0.14	−0.36 to 0.08	−0.17	−0.39 to 0.05
TAG score	1.51	0.47 to 2.55	1.52	0.49 to 2.55
Age 25+	−2.34	−12.28 to 7.60	−1.92	−11.84 to 8.00
Black, Asian and minority ethnicity	−3.37	−15.56 to 8.83	−4.93	−17.00 to 7.14
ADHD and/or ASD	−6.09	−16.29 to 4.10	−5.44	−15.63 to 4.75
At least one physical health problem	−6.46	−16.64 to 3.71	−6.30	−16.43 to 3.83
Female gender	4.47	−5.40 to 14.34	4.62	−5.21 to 14.45
Living in an adult social care setting (residential home or supported living)	−10.22	−20.47 to 0.35	−10.00	−20.14 to 0.14
Living independently	−7.31	−27.33 to 12.71	−7.17	−27.07 to 12.72
Working hours			9.43	−1.83 to 20.68

ABC-C, Aberrant Behavior Checklist-Community version 2; PAS-ADD, Psychiatric Assessment Schedule for Adults with Developmental Disabilities Checklist; SABS, Short Adaptive Behavior Scale; TAG, Threshold Assessment Grid; ADHD, attention-deficit hyperactivity disorder; ASD, autism spectrum disorder.

In step 1, working hours had a *P*-value <0.10 for the primary outcome and for some of the secondary outcomes (ABC-C irritability, lethargy/social withdrawal and hyperactivity/non-compliance subscales), so it was included in the multivariable analyses. However, for one of the secondary outcomes (inappropriate speech subscale), the exemptions and care coordination variables had a *P*-value <0.10 and therefore were included in the multivariable analysis for this secondary outcome.

For the primary outcome, change in ABC-C total score from baseline to 9-month follow-up, the TAG score, a measure of the severity of mental illness, was the only independent variable that was statistically significant (1.52, 95% CI 0.49–2.55). This was found in both the unadjusted and adjusted models.

As regards the secondary outcomes, change in the irritability subscale score at 9 months was significantly associated with living in an adult social care setting (−4.25, 95% CI −7.63 to −0.86) and the affective subscale of the PAS-ADD Checklist (4.44, 95% CI 0.59–8.29). Change in the hyperactivity subscale score was found to be significantly associated with the affective subscale of the PAS-ADD Checklist (4.65, 95% CI 0.46–8.83) in both the adjusted and unadjusted models.

Change in the ABC-C lethargy/social withdrawal subscale was found to be significantly associated with gender (2.98, 95% CI 0.04–5.93) when the model was adjusted for working hours.

There were no associations between the stereotypical behaviour and inappropriate speech subscales and demographic or clinical variables.

## Discussion

To our knowledge, there has not yet been any study examining the active ingredients of intensive support teams (ISTs) for adults with intellectual disabilities who display challenging behaviour. Prior to the IST-ID study, studies of IST service characteristics and patient outcomes had been conducted within individual ISTs, included small samples of ISTs or had been conducted within individual regions.^8,9,17–19^ Davison et al conducted a cross-sectional study that collected data from community teams that supported people with intellectual disabilities displaying challenging behaviour, but it did not investigate patient outcomes or any associations between teams and behaviour.^20^

We did not find any IST-level characteristics to be associated with changes in behaviour (improvement) as measured by the ABC-C total score. However, we saw a pattern emerge whereby participants’ clinical variables appeared to influence outcomes. The TAG score (a measure of the severity of mental illness), accommodation and affective status were significantly associated with change in primary and secondary measures of challenging behaviour.

Our results indicate that a higher TAG score at baseline is associated with an increase in ABC-C total score from baseline to 9-month follow-up. This association is supported by the well-documented relationship between mental illness and challenging behaviour in people with intellectual disabilities, although what mediates this relationship is unclear and likely to be multifaceted.^21–24^ Individuals with intellectual disabilities who are mentally unwell may display challenging behaviour as a secondary or atypical presenting feature.

The relationship identified between the affective subscale of the PAS-ADD Checklist and the irritability and hyperactivity subscales of the ABC-C may have arisen since those symptoms can be transdiagnostic and therefore present in many different mental disorders.^25–27^ Furthermore, the relationship identified between gender and the lethargy/social withdrawal subscale may be reflective of female gender having been identified as a risk factor for depression and mental ill health more generally among adults with intellectual disabilities.^28,29^

There are potentially several explanations as to why service-level features did not appear to influence patient outcomes in this study. First, it could be that individual characteristics are more important than service-level characteristics in underpinning outcomes, as has been found in a previous study of predictors of readmission in mental health services.^30^ Second, there may have been service-level characteristics that we did not measure and that may have been important in this context, such as area deprivation, intensity of support and specific input to crisis triage. Finally, it could be that the quality of care provided had a greater impact on patient outcomes and that this was not captured by the service-level characteristics we identified. For example, NICE and NHS England recommend that staff working within ISTs must be skilled and competent in delivering interventions to reduce risks associated with challenging behaviour and that these interventions should be delivered in a way that is person centred and is in line with relevant treatment manuals.^3,6,7,31^ In addition, NICE recommends that the clinical competency of staff should be regularly evaluated.^31^ These recommendations may be more difficult to measure, albeit they are an important factor in determining the clinical outcomes of patients supported by ISTs. A previous systematic review identified longer opening hours and inclusion of psychiatrists within the staff skill mix as central factors in implementing crisis resolution teams to prevent hospital admissions.^32^ However, in our study, our analysis did not support this.

### Limitations

It is important to highlight that this study has a number of limitations that must be considered when interpreting these findings. The independent variables included were binary and categorical and therefore may not have been sensitive enough to represent more subtle variation in how the ISTs were organised and structured. In addition, this secondary analysis focused on one clinical outcome, the ABC-C and its subdomain scores, although many other clinical outcomes may have been important indicators of service efficacy. It is also important to recognise that this was an exploratory study; although we sought to include the most clinically relevant service-level variables, we did not test any predetermined hypotheses.

## About the authors

**Lucretia Thomas** is a Foundation Year 1 Doctor at Homerton University Hospital, London, UK. **Brynmor Lloyd-Evans** is Professor of Mental Health and Social Inclusion, Division of Psychiatry, University College London, London, UK. **Louise Marston** is Professor of Clinical Trials Statistics, Department of Primary Care and Population Health, University College London, London, UK. **Angela Hassiotis** is Professor of Psychiatry of Intellectual Disability, Division of Psychiatry, University College London, London, UK.

## Supporting information

Thomas et al. supplementary material Thomas et al. supplementary material

## Data Availability

Anonymised data that support the findings of this study are available on request from the corresponding author, A.H.
