# An investigation into the critical ingredients of Intensive support teams for adults with intellectual disabilities who display challenging behaviour – ADDENDUM

**DOI:** 10.1192/bjb.2025.10196

**Published:** 2026-06

**Authors:** Lucretia Thomas, Brynmor Lloyd-Evans, Louise Marston, Angela Hassiotis

The above article was originally published with a supplementary file omitted. The supplementary file has now been published.
