# Supplementary material for: An investigation into the critical ingredients of intensive support teams for adults with intellectual disabilities who display challenging behaviour
Source: BJPsych Bull. 2025 Feb;49(1):5–10. doi: 10.1192/bjb.2023.94 (PMC11810469; doi:10.1192/bjb.2023.94)
Supplement: Thomas et al. supplementary material [file S2056469423000943sup001.docx]

# Items selected for data extraction from the IST-ID service level survey

- Q6: *Opening hours*
- Q6.1 *Accept phone referrals?* – working hours, extended hours, 24 hours, etc.
- Q9 – *Do you have a waiting list for your intensive support team? – Y/N*
- Q10 – *How long is the average waiting time?*
- Q12: *What professionals does your Intensive support team comprise of? Please indicate the number of people and the number of full time equivalent staff.*
- Q18: *What is the age range of service users accepted to your intensive support team?*
  - Lower limit, upper limit
- Q19: *What is the level of intellectual disability of service users accepted to your intensive support team? (Please tick all that apply)*
  - Profound
  - Severe
  - Moderate
  - Mild
- Q21: *Do you accept service users in contact with the criminal justice system?*
  - Yes/No
- Q22: *Does your intensive support team have any specific exclusion criteria?*
  - Yes/no
- Q23: *Does your intensive support team operate any exemption (e.g. people with ASD above the cut-off for intellectual disability)?*
  - Yes/no
- Q24: *Does your intensive support team work directly with people with intellectual disabilities who are displaying challenging behaviour?*
- Q26: *Does your intensive support team work with people with intellectual disabilities and challenging behaviour who are not in crisis but need support?*
- Q27 - Does your intensive support team provide any of the following services? (Please tick all that apply):
  - Specialist or enhanced/intensive support that works directly with people with intellectual disabilities who are transitioning out of hospital or experiencing other changes (e.g. moving accommodation, placement breakdown, or bereavement)?
  - Specialist or enhanced/intensive support that works directly with people with intellectual disabilities who are at risk of admission to hospital?
- Q34 *– Do all referrals for your intensive support ream have to be made through the duty line?*
- Q37 – *From which of the following sources will you accept referrals to your intensive support team?*
  - Referrals from a CLDT
  - Self-referrals from clients known to the team
  - Self-referrals from clients not known to the team
  - Referrals from family/friends/carers
  - GPs
  - Other primary care staff e.g. practice nurse, health visitor
  - NHS 111 service
  - Police
  - Other emergency services e.g. A&E
  - Third sector organisations e.g. day centres, housing services
  - Any other source
- Q38 *- Do you accept referrals without any further assessment (e.g. trusted assessors such as A & E liaison clinicians?)*
- Q39 – *How do you accept referrals?*
  - Phone
  - Letter
  - Email
  - In person
  - Other
- Q40 – *Does the team have a target response time within which to respond to a referrer with a decision about whether to see in person, after receiving a referral?*
  - Q41 – *what is the target maximum time (days)?*
- Q42 – *Do you have a target response time following a referral within which to commence an assessment in person by your team staff?*
  - Q43 – *What is the target maximum time (days)?*
- Q44 - *Do you have a target response time following a referral within which to complete an assessment in person by your team staff?*
  - What is the target maximum time (days)?
- Q46: does your assessment include a functional analysis of challenging behaviour? Y/N
- Q47 - Does your intensive support team provide the following interventions? (Please tick all that apply)
  - Positive Behaviour Support (including functional assessment)
  - Psychoeducational interventions with service-users’ family/paid carers
  - Other evidence based psychosocial therapies (e.g. anger management, mindfulness, intensive behavioural intervention, counselling, CBT, BSFT)
  - Assessment for activities of daily living (e.g. shopping, food preparation, cleaning, debt management)
  - Training for activities of daily living
  - Provide social and practical support for activities of daily living
- Q51: does your intensive support team operate ‘care co-ordination’ where individual clients have specific workers allocated to them?
- Q52: *Is there a minimum duration of visits to a service user from your team staff?*
  - Yes/no
- Q53: What is the average duration of a visit to a service user from your team?
  - <15 minutes
  - 15-30 minutes
  - 30-60 minutes
  - 60-120 minutes
  - 60-120 minutes
  - >120 minutes
- Q55*: How often will a service user typically be seen by your intensive support team?*
  - Less than once a week
  - Once a week
  - Twice a week
  - Three or more times a week
  - Other (please specify)
